# Towards simplified graph neural networks for identifying cancer driver genes in heterophilic networks

**DOI:** 10.1093/bib/bbae691

**Published:** 2025-01-03

**Authors:** Xingyi Li, Jialuo Xu, Junming Li, Jia Gu, Xuequn Shang

**Affiliations:** School of Computer Science, Northwestern Polytechnical University, Xi’an, 710072 Shaanxi, China; Research & Development Institute of Northwestern Polytechnical University in Shenzhen, Shenzhen, 518063 Guangdong, China; Faculty of Data Science, City University of Macau, Macau, 999078 Macau, China; School of Computer Science, Northwestern Polytechnical University, Xi’an, 710072 Shaanxi, China; Research & Development Institute of Northwestern Polytechnical University in Shenzhen, Shenzhen, 518063 Guangdong, China; School of Software, Northwestern Polytechnical University, Xi’an, 710072 Shaanxi, China; School of Software, Northwestern Polytechnical University, Xi’an, 710072 Shaanxi, China; School of Computer Science, Northwestern Polytechnical University, Xi’an, 710072 Shaanxi, China

**Keywords:** cancer driver genes, multi-omics data, graph neural networks, heterophilic networks, precision oncology

## Abstract

The identification of cancer driver genes is crucial for understanding the complex processes involved in cancer development, progression, and therapeutic strategies. Multi-omics data and biological networks provided by numerous databases enable the application of graph deep learning techniques that incorporate network structures into the deep learning framework. However, most existing methods do not account for the heterophily in the biological networks, which hinders the improvement of model performance. Meanwhile, feature confusion often arises in models based on graph neural networks in such graphs. To address this, we propose a Simplified Graph neural network for identifying Cancer Driver genes in heterophilic networks (SGCD), which comprises primarily two components: a graph convolutional neural network with representation separation and a bimodal feature extractor. The results demonstrate that SGCD not only performs exceptionally well but also exhibits robust discriminative capabilities compared to state-of-the-art methods across all benchmark datasets. Moreover, subsequent interpretability experiments on both the model and biological aspects provide compelling evidence supporting the reliability of SGCD. Additionally, the model can dissect gene modules, revealing clearer connections between driver genes in cancers. We are confident that SGCD holds potential in the field of precision oncology and may be applied to prognosticate biomarkers for a wide range of complex diseases.

## Introduction

In biomedical research, it is generally accepted that the onset of cancer is associated with the accumulation of mutations in driver genes. Therefore, accurately identifying cancer driver genes is paramount for clarifying the biological processes that underlie carcinogenesis and for the advancement of personalized oncology pharmaceuticals [1, 2].

Over the past decade, many statistics methods have emerged to detect cancer driver genes. The approaches based on frequency such as MuSic [3], MutSigCV [4], and OncodriveCLUST [5] generally operate under the assumption that mutations in driver genes tend to recur more often across samples compared to those in non-driver genes, thereby facilitating the recognition of highly mutated genes as cancer driver genes. Nonetheless, these techniques often struggle to identify driver genes with rare mutations.

As machine learning (ML) advances rapidly and large-scale cancer genomics projects involving thousands of patients continue to release multi-omics data [6–8], numerous ML-based approaches have also shown noteworthy success in identifying cancer driver genes. Essentially, these methods concentrate on deriving low-dimensional representations of genes from a variety of biological attributes to effectively distinguish driver genes from non-driver genes. For instance, LOTUS [9] employs a support vector machine to detect pan-cancer driver genes, while TUSON [10] utilizes a LASSO regression for the same purpose. Furthermore, there are various methods designed to detect personalized cancer driver genes, such as sysSVM [11] and IMCDriver [12]. Nonetheless, most existing ML-based methods focus exclusively on multi-omics data to construct gene embeddings for the identification of cancer driver genes, failing to take into account the topological features provided by biological networks.

Some methods focusing on networks are conducted under the assumption that cancers are driven by alterations in numerous genes that interact intimately within biological networks. Therefore, these methods identify cancer driver genes by targeting genes that take on critical topological positions in biological networks using network propagation strategies, such as pgWalk [13], RWRH [14], and BiRW [15]. However, the lack of omics data has weakened their performance.

With the emergence of graph neural networks (GNNs), the fusion of multi-omics data and networks for identifying cancer driver genes is becoming promising [16–19]. For example, EMOGI [20] is a method built on graph convolutional neural network (GCN) [21] that leverages multi-omics data as gene features along with the protein–protein interaction (PPI) network to identify cancer driver genes. MTGCN [22] merges biological and structural features to develop enhanced representations, utilizing a multi-task framework designed to improve the tasks of node and link prediction. SMG [23] performs a node reconstruction task to obtain a pre-trained encoder, which is then used for downstream tasks including gene identification and disease subnetwork identification. Although the aforementioned methods have yielded some success in cancer driver gene identification, they all rely on the homophily assumption. Recently, many researches have shown that most biological networks tend to exhibit heterophily property [24, 25]. For instance, PPIs encompass both physical interactions and functional associations between various biomolecules, exhibiting a low homophily ratio, as elaborated in Supplementary Materials, Section 2. Additionally, the quantity of cancer driver genes is markedly fewer than the total number of genes within the biomolecular networks [26, 27].

A few methods have tried to enhance the accuracy of identifying cancer driver genes on heterophilic graphs. For example, HGDC [27] is designed to tackle the heterophilic nature of biological networks to identify cancer driver genes, integrating graph diffusion technique with hierarchical attention mechanisms. However, the graph diffusion convolution (GDC) employed by HGDC necessitates the calculation and storage of diffusion matrices such as the heat diffusion kernel or personalized PageRank, which can be computationally burdensome for large-scale graphs. Moreover, based on GCN, HGDC also encounters the issue of feature confusion, a common challenge for traditional GNNs in heterophilic graphs.

In this study, we propose an efficient approach named SGCD (Simplified Graph neural network for identifying Cancer Driver genes in heterophilic networks), which utilizes simplified GNNs for identifying cancer driver genes in heterophilic networks. We innovatively introduce representation separation (RS) to replace the traditional message-passing mechanism of GCN, effectively mitigating the issue of feature confusion in GNNs when dealing with these graphs. The experimental findings consistently indicate that SGCD surpasses the state-of-the-art approaches, highlighting its excellence. Furthermore, subsequent model interpretability experiments and biological interpretability experiments provide compelling evidence for the powerful interpretability of SGCD. In addtion, SGCD can dissect gene modules, enabling a comprehensive analysis of the association between gene modules and cancer mechanisms.

## Material and methods

### Data collection and preprocessing

From the Cancer Genome Atlas (https://portal.gdc.cancer.gov/), we collect oncogenomics (mutations and copy number variations), epigenomics (DNA methylation), and transcriptomics (gene expression) data, comprising over 29 446 samples across 16 distinct malignancies.

For each gene, we calculate gene mutation rate, copy number aberrations, differential DNA methylation rate, and differential gene expression rate across the 16 cancer types (see Supplementary Materials Section 3 for details). By integrating the feature vectors from all cancer types, we construct a 64-dimensional feature vector for every gene. Subsequently, min-max normalization is conducted on the features of each gene.

The lists of known driver genes are sourced from the Network of Cancer Genes (NCG) v6.0 [28], COSMIC Cancer Gene Census (CGC v91) [26], and DigSEE [29], which serve as positive samples. In contrast, negative samples are obtained by excluding gene lists from NCG, COSMIC, OMIM [30], as well as pathways from KEGG [31].

The PPI data is from CPDB [32], MULTINET [33], PCNet [34], STRINGdb [35], and IRefIndex [36]. Particularly, we exclusively consider interactions with high confidence. For the CPDB network, only interactions with a confidence score above 0.5 are included, while for STRINGdb, a threshold of 0.85 is applied. MULTINET and the 2015 IRefIndex version are directly retrieved from the Hotnet2 github repository. For the updated IRefIndex, our primary focus is on binary interactions between two proteins as well as human interactions. The process of PCNet is the same as EMOGI [20]. To integrate different PPI data into the consistent format, we first convert gene names from different formats into uniform symbol names. Each gene is then characterized as a node in the graph, with edges constructed between nodes to reflect the corresponding protein-protein interactions. Consequently, we obtain a total of six PPIs in a unified format, and the overview of PPIs can be found in Table S2 in the Supplementary Materials.

### Overview of SGCD

**Figure 1:**
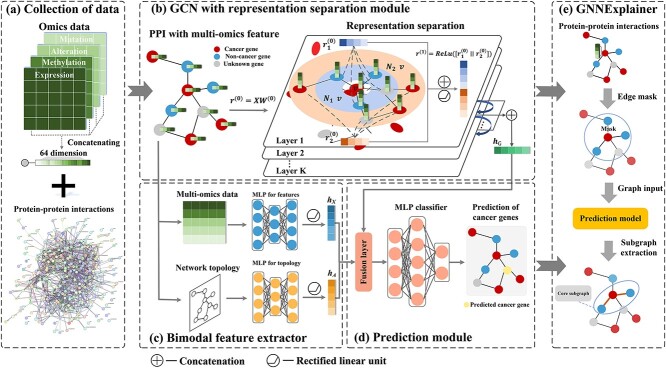
Overview of SGCD. (a) Collection of PPIs and 64-dimensional multi-omics data. (b) GCN with RS Module. We leverage a GCN with RS to learn node embeddings from multi-omics and PPI networks. (c) Bimodal feature extractor. It incorporates two Multilayer Perceptrons (MLP) to separately embed the topological information and the multi-omics. (d) Prediction module. We combine the convolution-derived representation with the bimodal MLP-derived representation through a linear layer to estimate whether a gene serves as a driver gene. (e) GNNExplainer is adopted to identify cancer gene modules.

SGCD is a straightforward and effective method comprising primarily two components: a GCN with RS and a bimodal feature extractor. The overview of SGCD is shown in Fig. 1. Firstly, SGCD leverages a GCN with RS to learn node embeddings from multi-omics and PPIs. Secondly, SGCD incorporates a bimodal feature extractor to preserve the topological information from PPIs and the multi-omics information. Finally, SGCD aggregates the representations obtained from the above two modules to calculate the likelihood of a gene functioning as a driver gene. Additionally, SGCD employs GNNExplainer to identify cancer gene modules by detecting compact subgraph structures through a masking method.

### GCN with RS

Recently, many studies have theoretically demonstrated that RS can enhance the generalization capability of GNNs on heterophilic graphs [37, 38]. Therefore, SGCD introduces RS to replace the traditional message-passing mechanism of the GCN. As far as we know, we are the first to utilize RS within the framework for identifying cancer driver genes. Considering there is an attributed biological network denoted as $\mathcal{G}=\{A,X\}$. $A$ represents the adjacency matrix and $X \in \mathbb{R}^{n\times f}$ is the features matrix, where $n$ indicates the overall number of genes and $f$ is the dimension of multi-omics data. Firstly, to prevent significant differences in features distribution between high-degree and low-degree nodes during information passing, SGCD applies symmetric normalization as follows: 


(1) \begin{align*} & \tilde{A}=\tilde{D}^{-\frac12}(A+I)\tilde{D}^{-\frac12}\end{align*}

where $\tilde{D}=D+I$, $D$ is the diagonal degree matrix of $A$, and $I$ is the identity matrix.

To fully leverage node representations, SGCD extracts both the representations of first-order neighboring nodes and second-order neighboring nodes. Besides, since potentially large differences in features between connected nodes in heterophilic graph, aggregating neighbor information through layer-by-layer stacking (i.e., sum operation) can cause node representations to become similar, which hinders the performance of the model from effectively distinguishing between different classes of nodes [39]. SGCD alleviates the confusion in representing central nodes in dissimilar node pairs through concatenation. The node representation $h_{G}$ can be defined as follows: 


(2) \begin{align*} r_{1}^{(l)}&=\tilde{A}r^{(l)} \end{align*}

(3) \begin{align*} r_{2}^{(l)}&=\tilde{A}^{2}r^{(l)} \end{align*}

(4) \begin{align*} r^{(l+1)}&=\mathrm{ReLU}(\mathrm{concat}([r_{1}^{(l)},r_{2}^{(l)}])) \end{align*}

(5) \begin{align*} h_{G}&=\mathrm{log\sigma}(\mathrm{concat}([r^{(1)}W^{(1)},..., r^{(l)}W^{(l)},..., r^{(L)}W^{(L)}])) \end{align*}

where $r^{(0)}=XW^{(0)}$, $l \in \{0,1,...,L\}$, $L$ is the layer number of convolutions, $\{ W^{(0)},...,W^{(l)},...,W^{(L)} \}$ are a series of trainable matrices, $\mathrm{ReLU}$ is the rectified linear unit, $\sigma $ is softmax function.

### Bimodal feature extractor

Due to the complex relationships between graph topology and label distributions in heterophilic graphs, many conventional GNNs may fail to fully leverage the graph topology in such contexts [40, 41]. In contrast, MLPs are essentially fully connected neural networks, where each layer performs a linear transformation on the feature vectors followed by a nonlinear activation function. This design makes MLPs rely solely on the features of the nodes without considering adjacency relationships, thereby maintaining local independence of nodes and effectively avoiding interference from neighboring nodes. In our study, regarding $\mathcal{G}$ as a combination of two modalities: topology and multi-omics, SGCD designs a bimodal feature extractor based on MLP to separately embed the adjacency matrix into $h_{A}$ and the multi-omics into $h_{X}$. This design enables SGCD to acquire distinct topological and omics information, avoiding a conflation of them. Then, SGCD integrates information from two modalities to generate the representations of nodes. The details are as follows: 


(6) \begin{align*} & h_{A}=\mathrm{MLP_{A}}(A) \end{align*}

(7) \begin{align*} & h_{X}=\mathrm{MLP_{X}}(X) \end{align*}

(8) \begin{align*} & h_{BI}= \alpha h_{A} + \beta h_{X} \end{align*}

where $\alpha $ and $\beta $ are hyperparameters.

### Model prediction

Ultimately, we combine the convolution-derived representation $h_{G}$ with the bimodal MLP-derived representation $h_{BI}$ through a linear layer to calculate the likelihood of a gene functioning as a driver gene as follows: 


(9) \begin{align*}& p=\mathrm{\log\sigma}[(h_{G}+ h_{BI})W^{^{\prime}}]\end{align*}

where $W^{^{\prime}}$ is a trainable matrix.

In our study, we utilize the binary cross-entropy loss to train the model: 


(10) \begin{align*}& L=-\sum_{i=1}^{n} \left(y_{i} \log\left(p_{i}\right)\right) + (1-y_{i}) \log\left(1-p_{i}\right)\end{align*}

where $y_{i}$ is the true label of gene $i$, $p_{i}$ is the prediction score of gene $i$, and $n$ is the number of nodes.

Our model is built using Python 3.8, PyTorch Geometric 2.0.1 [42] and PyTorch 1.10.1. To identify the optimal hyperparameters for SGCD, we apply Optuna [43] to automate hyperparameter search. This optimization is obtained using stratified five-fold cross-validation on the training set, ensuring consistent proportions of known cancer genes and non-cancer genes across all sets. In our experiments, the hidden layer dimension of GCN with RS is set to 64. To regularize the model and mitigate overfitting, a weight decay of $5\times 10^{-5}$ is applied. The learning rate is set to 0.0149. The coefficient for ${\text{MLP}}_{\text{A}}$ is 0.0204, and for ${\text{MLP}}_{\text{X}}$ it is 0.001. The model is trained for 30 epochs, and the number of hops is 2.

### GNNExplainer

We apply GNNExplainer [44] to interpret key interactions for genes. For a given node $i$, GNNExplainer identifies a connected subgraph ${G_{Si} \subseteq G}$. It is described below: 


(11) \begin{align*}& \min_{M_{i}, f_{i}}-\log P_{\Phi}\left(Y=\hat{y}_{i} \mid G=A_{i} \odot \sigma\left(M_{i}\right), X=\boldsymbol{X}_{i} \odot \sigma\left(f_{i}\right)\right)\end{align*}

where $A_{i}$ represents the adjacency matrix and $X_{i}$ represents multi-omics features. $M_{i} \in \mathbb{R}^{n \times n}$ denotes the mask matrix for the adjacency matrix and ${f_{i} \in \mathbb{R}^{m}}$ denotes multi-omics features, which need to be learned for node $i$. $n$ is the total number of genes, and $m$ denotes the dimension of the multi-omics features. The trained GNN model is denoted by $\Phi $. $\odot $ stands for Hadamard product, and $\sigma $ refers to the activation function (sigmoid).

For undirected graphs like PPI, $M_{i}$ is preserved while optimizing. The values of $M_{i}$ reflect the significance of the associated edges. The explanatory subgraph $G_{Si}$ used for predicting $\hat{y}_{i}$ at node $i$ is determined as follows: 


(12) \begin{align*}& G_{Si} = A_{i} \odot \mathbf{1}\left\{ M_{i} \geq \theta \right\}\end{align*}

where $\theta $ stands for edge threshold.

## Results

### Performance assessment of SGCD

To assess the effectiveness of SGCD in cancer driver gene identification, we compare it with five methods, including the standard GNN method GCN [21] and four advanced GCN-based approaches specifically designed for cancer driver gene identification, including EMOGI [20], MTGCN [22], SMG [23], and HGDC [27].

GCN [21] is a quintessential type of GNN that aggregates features from itself along with features from its direct neighbors. This mechanism captures the local information in the graph, allowing for richer node representations.
EMOGI [20] is an explainable method built on GCN that utilizes pan-cancer multi-omics data as gene features in combination alongside the PPI networks to generate more meaningful representations.
MTGCN [22] merges biological and structural features to develop enhanced representations. It introduces a Chebnet-based multi-task framework [45], boosting both the main and auxiliary tasks. In addition, it includes a weight learning mechanism that dynamically adjusts the relative contributions of each task.
SMG [23] adopts the pretrain-finetune strategy. During the pretraining phase, it randomly masks certain nodes, and utilizes an GNN-based encoder to recover these masked nodes by referring to the information from their surrounding neighbors. During the subsequent fine-tuning stage, SMG takes advantage of the pre-trained encoder to represent the PPIs and utilizes a tailored layer to predict results for the specific tasks.
HGDC [27] is designed to tackle heterophily within biological networks for the identification of driver genes. HGDC combines graph diffusion methods with hierarchical attention mechanisms. By utilizing graph diffusion to create supplementary views, HGDC significantly improves prediction accuracy across various biological networks.

**Figure 2:**
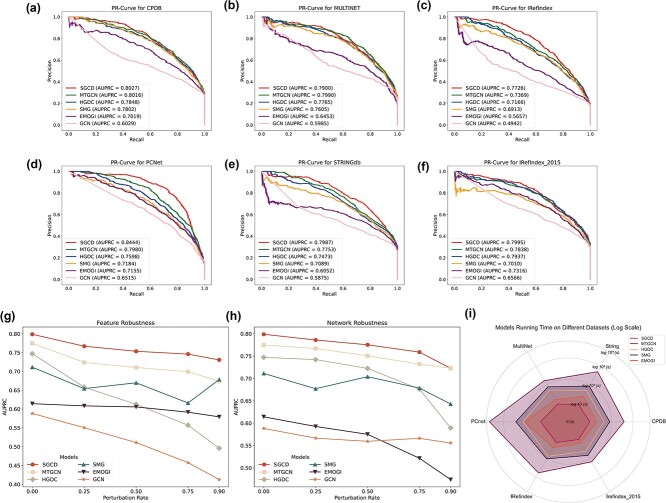
Performance assessment of SGCD. (a) AUPRC performance comparison on CPDB. (b) AUPRC performance comparison on MULTINET. (c) AUPRC performance comparison on IRedfIndex. (d) AUPRC performance comparison on PCNet. (e) AUPRC performance comparison on STRINGdb. (f) AUPRC performance comparison on IRedfIndex_2015. (g) Feature robustness analysis of SGCD and baseline models. (h) Network robustness analysis of SGCD and baseline models. (i) The time overhead analysis of SGCD and baseline models.

To guarantee an equitable comparison, all methods utilize identical processed feature vectors and PPI networks, including CPDB [32], MULTINET [33], PCNet [34], STRINGdb [35], IRefIndex [36], and IRefIndex-2015 [36]. The parameters for the baseline models are either configured according to their papers or adjusted as needed to maximize their performance. The datasets are divided into a training set (75%) and a testing set (25%), and then we train the SGCD and baseline models separately to attain optimal performance. As shown in Fig. 2(a) to Fig. 2(f), SGCD achieves the best AUPRC in different PPIs than other baseline models, indicating the advantage of SGCD in detecting potential cancer driver genes.

To validate the robustness of SGCD, we evaluate performance under both feature perturbation and network perturbation. Feature perturbation is implemented by randomly masking node features, where the values are set to 0, with masking rates of 0.25, 0.50, 0.75, and 0.90. Network perturbation is performed by randomly deleting edges in the network, with deletion rates set at 0.25, 0.50, 0.75, and 0.90. Then, we train SGCD and baseline models at each perturbation rate. The results shown in Fig. 2(g) and 2(h) demonstrate that with the increasing rate of feature perturbation or network perturbation, SGCD consistently shows the best performance and experiences a relatively smaller decrease in performance compared to baseline models. These results indicate that SGCD evinces strong robustness, adapts well to real-world conditions, and consistently performs with stability and excellence.

The time overhead of an algorithm is a crucial metric for evaluating its efficiency. In this research, we record the running time of SGCD and baseline models across different PPIs. As shown in the Fig. 2(i), SGCD persistently achieves minimal time overhead (log 10-transformed) across all datasets, highlighting its exceptional time efficiency.

### Ablation study

To analyze the contribution of every step within SGCD model architecture, we carry out ablation experiments on MULTINET. The results of ablation experiments are shown in Table 1. We notice that the performance of merely using classic MLP architecture (${\text{MLP}}_{\text{A}}$ + ${\text{MLP}}_{\text{X}}$) significantly surpasses that of GCN (as shown in Fig. 2a), which suggests that traditional convolutional methods may lead to feature confusion, resulting in even worse performance than MLP. Additionally, the comparison between GCN with RS and the GCN-based method EMOGI (as shown in Fig. 2a) also demonstrates that feature separation can effectively reduce the confusion in node information aggregation within heterophilic networks, thereby significantly improving the performance of models in such networks. Finally, the complete framework of SGCD model shows superior performance compared to GCN with RS + ${\text{MLP}}_{\text{A}}$ or GCN with RS + ${\text{MLP}}_{\text{X}}$, indicating that SGCD successfully improves cancer driver gene identification by integrating GCN with RS and bimodal feature extractor.

**Table 1:** The ablation results of SGCD

Model	AUPRC	AUROC	F1	ACC
GCN with RS + ${\text{MLP}}_{\text{A}}$	0.7881	0.9320	0.6917	0.9015
GCN with RS + ${\text{MLP}}_{\text{X}}$	0.7560	0.9242	0.6648	0.8948
GCN with RS	0.7557	0.9242	0.6634	0.8946
${\text{MLP}}_{\text{A}}$ + ${\text{MLP}}_{\text{X}}$ (bimodal feature extractor)	0.6021	0.7791	0.5288	0.8755
GCN with RS + ${\text{MLP}}_{\text{A}}$ + ${\text{MLP}}_{\text{X}}$ (SGCD)	0.7900	0.9324	0.6939	0.9021

Overall, the results of the ablation study confirm the effectiveness of each step of SGCD. Specifically, the results demonstrate that substituting RS for the traditional message-passing mechanism of GCN significantly reduces feature confusion, ultimately improving the generalization ability of GNNs in heterophilic graphs. Furthermore, given the complex information between graph topology and label distribution in heterophilic graphs, the use of bimodal feature extractor can effectively extract distinct topological and omics information from these graphs, thus enabling a more optimized utilization of graph information.

### Performance on independent test sets

**Figure 3:**
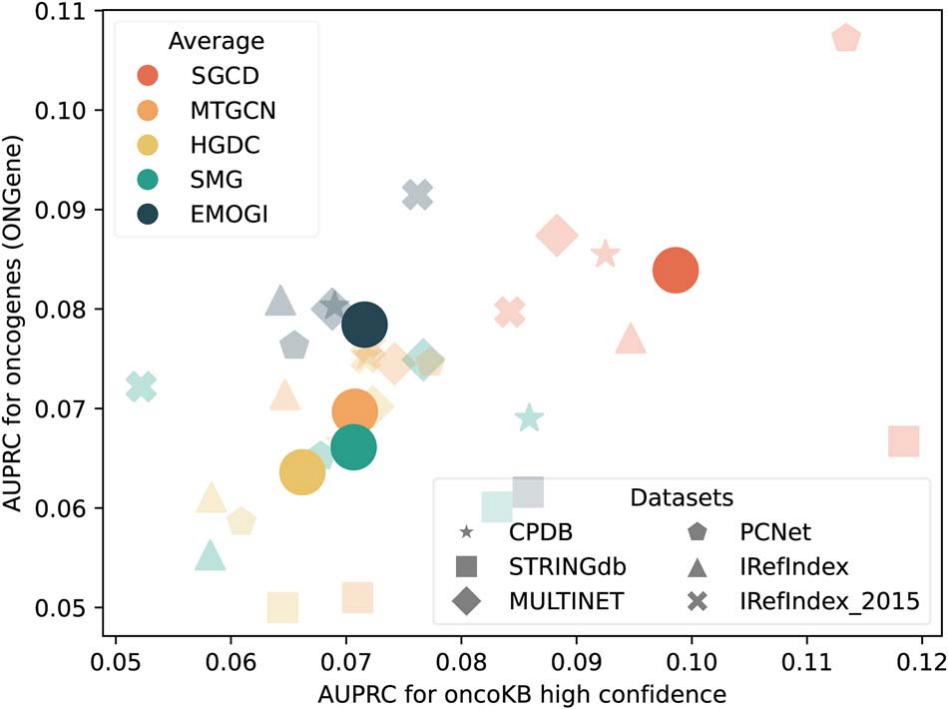
Performance of SGCD and baseline models across two independent sets based on OncoKB and ONGene.

To verify if the performance of SGCD and baseline models are biased towards any cancer-related datasets, we evaluate them on two independent datasets. We train SGCD the baseline methods using labeled samples, including both positive and negative instances. The trained models are next used to predict cancer-related genes in two independent test sets: one consists of 320 genes from the OncoKB [46] and another with 388 genes from the ONGene [47]. After excluding genes that overlap with the training samples, we regard the predicted cancer driver genes in the test set as true positives, while genes absent from the test set are classified as false positives. Although all methods perform relatively poorly due to the insufficient number of true positives in the independent test sets, Fig. 3 shows that SGCD consistently surpasses the baseline models on both OncoKB and ONGene.

### Prediction of potential cancer driver genes

**Figure 4:**
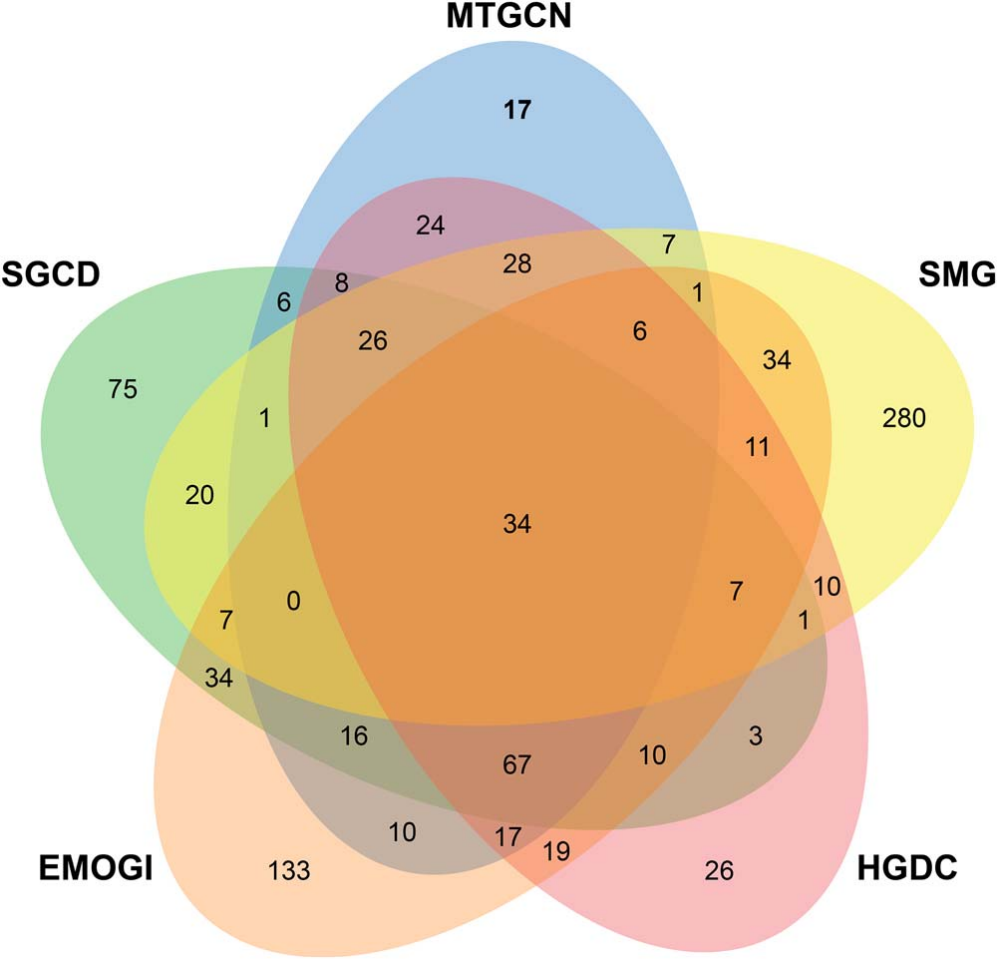
Venn diagram of the overlap between the SGCD and other driver genes identification methods.

We use SGCD to train and predict on six PPIs. Then, by aggregating the top 100 predicted driver genes from each PPIs, a list of 315 potential cancer driver genes is obtained, as demonstrated in Table S1 in the Supplementary Materials. Comparing SGCD with several other identification methods, we observe that SGCD can predict unique driver genes not observed by the other methods, as shown in Fig. 4. This underscores the capability of SGCD to uncover overlooked driver genes, highlighting its valuable genetic insights. Among these unique genes, most of them are linked to cancer onset and development. For example, many studies have demonstrated that GNB1 is involved in the progression and drug resistance of multiple cancer types [48]. Extensive research has demonstrated that NR2C2 potentially function as either a tumorigenic gene or a tumor-suppressive gene, depending on the type of tumors [49, 50]. A previous study has indicated that PPP2CA has potential to serve as a tumor suppressor gene across various cancers, with its expression potentially modulated by rs13187105 or other SNPs that exhibit strong linkage disequilibrium [51].

To further analyze these potential cancer genes, we compare them with two sets of candidate cancer driver genes derived from sources based on published literature. The first source is the CancerMine [52], a text-mined and regularly updated resource that catalogs drivers, oncogenes and tumor suppressor genes across various cancer types. The second source consists of a highly reliable gene set gathered from the Candidate Cancer Gene Database (CCGD) [53], encompassing all available data from transposon-based forward genetic screens related to cancer. Overall, approximately 91% (287/315) of the potential driver genes have evidences supporting their association with cancer. Furthermore, among these evidence-supported genes, over 88% (253/287) are supported by CancerMine, over 76% (220/287) are supported by CCGD and over 64% (186/287) are supported by both CancerMine and CCGD. These experimental results further substantiate the strong reliability of the cancer driver genes identified by SGCD.

### Enrichment analysis

**Figure 5:**
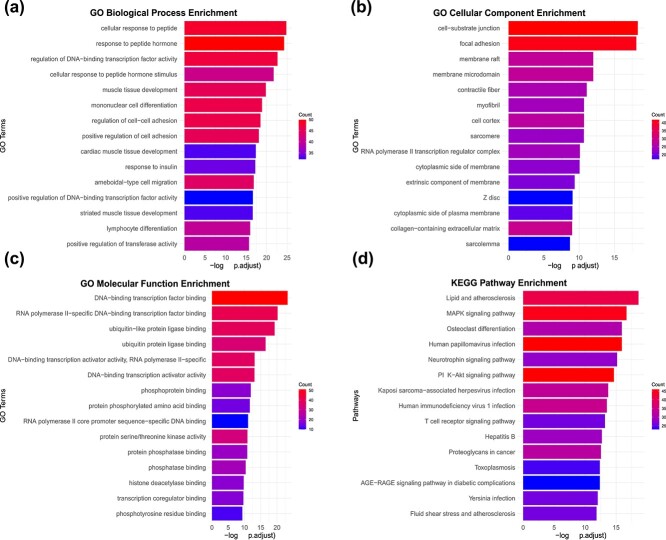
Enrichment analysis. (a) Go biological process enrichment. (b) GO cellular component enrichment. (c) Go molecular function enrichment. (d) KEGG pathway enrichment.

We perform enrichment analysis of Gene Ontology (GO) and KEGG pathways on the predicted cancer driver genes, and the results show that these predicted genes exhibit notable enrichment in numerous cancer-related pathways. For instance, as shown in Fig. 5(a), in Go biological process enrichment, cell–cell adhesion is essential for enabling extravasation from the primary tumor and subsequent metastasis, while the loss of cell adhesion molecules is closely linked to tumor progression [54]. As shown in Fig. 5(b), in cellular component enrichment, membrane rafts, as targets for cancer treatment, significantly contribute to cell survival regulation by enhancing Akt activation. They are closely tied to their pivotal role in regulating multiple stages of malignant cell transformation, including growth, apoptosis susceptibility, invasiveness, and metastatic capacity [55]. As shown in Fig. 5(c), in GO molecular function enrichment, phosphatases act as molecular switches capable of activating or deactivating various signaling pathways, leading to abnormal cellular activities such as unchecked proliferation, differentiation, angiogenesis, and metastasis. Numerous phosphatases have been associated with the initiation and pathogenesis of various types of cancer [56]. As shown in Fig. 5(d), in KEGG pathway enrichment, the PI3K-Akt signaling pathway, a pivotal regulator of diverse cellular functions, is frequently dysregulated in cancer, fostering tumor initiation and progression. Targeting this pathway, either as a standalone approach or in conjunction with other therapeutic modalities, has emerged as a highly efficacious strategy in the battle against cancer [57].

### Drug sensitivity analysis

**Figure 6:**
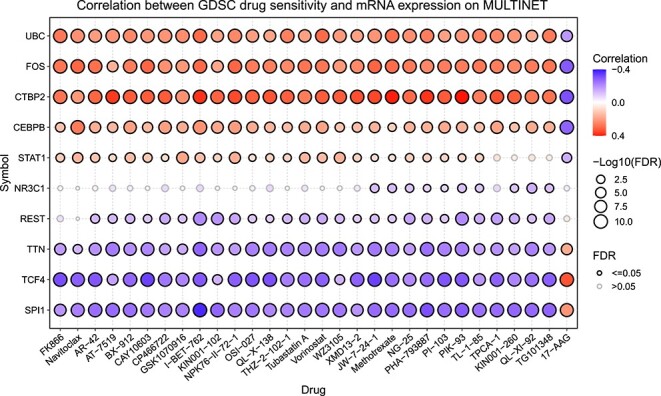
Correlation between drug sensitivity and mRNA expression for the top 10 predicted cancer driver genes.

Given that drug sensitivity reveals how cancer driver genes influence cancer cell responses to specific drugs, we select the top 10 predicted cancer driver genes in each PPIs for Cancer Therapeutics Response Portal drug sensitivity analysis using Gene Set Cancer Analysis (http://bioinfo.life.hust.edu.cn/GSCA) [58, 59]. Figure 6 illustrates the drug sensitivity analysis results for MULTINET, while the results of other PPIs are present in Fig. S1 in the Supplementary Materials. The results of the drug sensitivity analysis demonstrate that cancer driver genes identified by SGCD has the potential to provide important perspectives on drug targets, thereby enhancing both the effectiveness and precision of cancer treatment. As exemplified by MULTINET in Fig. 6, the majority of these genes exhibit significant correlations with drug sensitivity, highlighting their potential involvement in affecting responses to particular cancer therapies. For instance, AR-42 is an innovative histone deacetylase inhibitor, and it demonstrates antitumor effects in pancreatic cancer cells by impacting various biochemical pathways [60]. PIK-93 promotes a treatment-friendly tumor microenvironment when used in conjunction with anti–PD-L1 antibodies, thus improving the effectiveness of PD-1/PD-L1 blockade cancer immunotherapy [61]. FK866, by blocking NAMPT-driven NAD+ production, can reduce the activation and stemness of CAFs, diminish the release of inflammatory cytokines and chemokines by suppressing PITX3 expression, and thereby inhibit colorectal cancer metastasis [62].

### Gene module dissection in pan-cancer

We utilize the GNNExplainer [44] to elucidate the influential factors associated with cancer driver genes across multi-omics data, subsequently detecting the cancer gene modules. GNNExplainer [44] utilizes a masking strategy to maximize the mutual information between the predictions and the distribution of potential subgraph structures, thereby identifying the most compact gene module.

**Figure 7:**
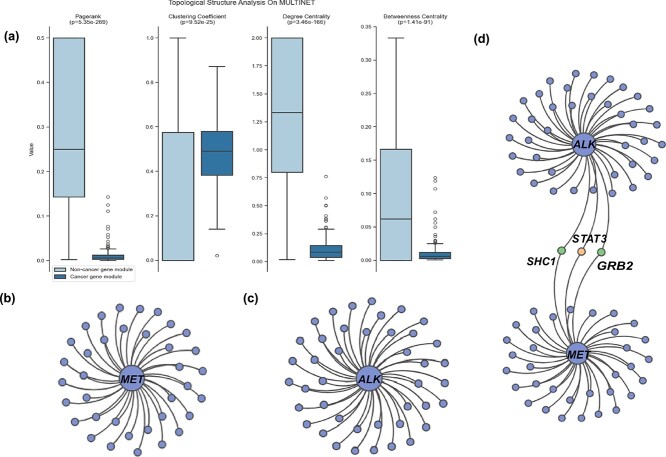
Cancer gene module analysis on SGCD. (a) Graphical metrics of gene modules. (b) The structure of MET gene module. (c) The structure of ALK gene module. (d) high-order gene module of MET and ALK.

Firstly, we compare the topological characteristics of cancer gene modules with non-cancer gene modules using graphical metrics, such as PageRank, clustering coefficient, degree centrality, and betweenness centrality (see Supplementary Materials Section 5 for details). These modules are obtained by separately applying GNNExplainer to the predicted cancer driver genes and the non-cancer driver genes. The results of MULTINET are shown in Fig. 7, which demonstrate that the topological structures of cancer gene modules exhibit significantly greater consistency compared to those of non-cancer gene modules (*P* < 9.52e-25, *t*-test). The results of other PPIs can be found in the Fig. S2 in the Supplementary Materials.

Furthermore, we analyze the relationship between different gene modules. In lung cancer, MET and ALK are the most commonly encountered driver genes [63], but they have different carcinogenic mechanisms. MET gene abnormalities primarily include MET exon 14 mutations, MET amplification, MET gene fusions, and MET protein overexpression. These alterations can result in abnormal activation of the MET signaling pathway, which, in turn, can drive tumor development and progression [64]. By contrast, the majority of mutations in the ALK gene occur as translocations with a partner gene, resulting in a fusion oncogene, which is subsequently overexpressed in cancers [65]. The gene modules of MET and ALK identified by our method are shown in Fig. 7(b) and 7(c). Moreover, we notice that the MET gene module and the ALK gene module share three common genes: STAT3, SHC1, and GRB2, which combine the two gene modules into a high-order gene module as shown in Fig. 7(d). It is worth noting that among these genes, STAT3 is the known cancer driver gene, while SHC1 and GRB2 are cancer driver genes predicted by SGCD (ranked in the top 50 predicted cancer driver genes of MULTINET). The research has shown that cancer driver genes are often found together in the same modules instead of being randomly scattered [66], and this suggests that the identified cancer genes SHC1 and GRB2 are promising candidates for cancer driver genes.

## Conclusion

Nowadays, cancer is one of the major threats to human health, and its underlying mechanisms are complex. It is widely accepted in the biomedical field that cancer emerges due to the accumulation of mutations across various genes. Therefore, cancer driver gene identification is important for uncovering the processes of cancer initiation and progression.

In this research, we present an innovative model, SGCD, which employs simplified GNNs to identify cancer driver genes in heterophilic networks. A key innovation of SGCD is the introduction of the GCN with RS module, where RS replaces the traditional message-passing mechanism, effectively mitigating potential feature confusion issues inherent in conventional GNNs. Additionally, SGCD utilizes a bimodal feature extractor to capture both topological and omics information, thereby enhancing identification performance. The experimental findings indicate that SGCD surpasses the state-of-the-art approaches, strengthening the predictive accuracy and robustness of the model. Furthermore, subsequent model interpretability experiments and biological interpretability experiments reveal that the potential cancer driver genes identified by SGCD are closely associated with cancer, validating the strong interpretability of SGCD. In addition, the model is capable of dissecting gene modules, providing deeper insights into the relationships between genes and their roles in cancer. We believe that SGCD is a general method, offering novel perspectives on the identification of cancer driver genes and allowing its application beyond the field of cancer genomics to other complex diseases.

**Key Points**

- An efficient method SGCD is developed for identifying cancer driver genes in heterophilic network by utilizing simplified graph neural networks.
- SGCD introduces representation separation to replace the traditional message-passing mechanism in GCN, significantly mitigating potential feature confusion.
- The computation experiments show the superiority of SGCD compared to other baseline models.
- The biological interpretability experiments provide compelling evidence supporting the reliability of SGCD.
- SGCD can dissect gene modules, revealing clearer connections between driver genes in cancers.

## Supplementary Material

SGCD_supplementary_bbae691

## Data Availability

All data is publicly available and the code of SGCD can be freely downloaded from https://github.com/xingyili/SGCD.
