# Supplementary material for: Towards simplified graph neural networks for identifying cancer driver genes in heterophilic networks
Source: Brief Bioinform. 2025 Jan 3;26(1):bbae691. doi: 10.1093/bib/bbae691 (PMC11697181; doi:10.1093/bib/bbae691)
Supplement: SGCD_supplementary_bbae691 [file sgcd_supplementary_bbae691.pdf]

Towards Simplified Graph Neural Networks for Identifying Cancer Driver Genes in Heterophilic Networks

Xingyi Li[1,3,4,*], Jialuo Xu[1], Junming Li[2,3], Jia Gu[4] and Xuequn Shang[1,*]

[1]School of Computer Science, Northwestern Polytechnical University, Xi'an, Shaanxi, 710072; [2]School of Software, Northwestern Polytechnical University, Xi'an, Shaanxi, 710072; [3]Research & Development Institute of Northwestern Polytechnical University in Shenzhen Shenzhen, 518063, China; [4]Faculty of Data Science, City University of Macau, Macau, 999078, China.

1. Potential cancer driver genes

Table S1.: Potential cancer driver genes

1	TP53BP1	2	EEF1A1	3	MEIS1	4	MDC1	5	PRKCA	6	UBE2I	7	ARRB1	8	PRKCZ	9	HIST1H3F	10	BMP4
11	VCAM1	12	SOS1	13	HSPA1B	14	CALM2	15	PPARGC1A	16	PLCG2	17	TNFRSF1A	18	UBB	19	CUBN	20	CHEK1
21	TERF1	22	COL11A1	23	PRPF8	24	HIST1H3D	25	PPP2R1B	26	GNB1	27	DYNC1H1	28	KDM5B	29	TGFB2	30	MYLK
31	SMARCC2	32	FANCM	33	TF	34	PAXIP1	35	ACTN2	36	RELB	37	PIK3CG	38	RPS27A	39	VCP	40	ACTB
41	APP	42	RELN	43	LRP1	44	FGF9	45	TAB2	46	NCOA6	47	IGF2R	48	PIK3CD	49	YAP1	50	CDH2
51	RARB	52	PPP1CA	53	MAP3K5	54	TRIM28	55	NFKB1	56	HDAC1	57	DSP	58	PTPRJ	59	TCF4	60	ZBTB7A
61	DNM1	62	LYN	63	EGLN3	64	NR2F2	65	NGF	66	TOP2A	67	RIMS2	68	HIST1H3H	69	WNT2	70	NEB
71	WNT5A	72	ANXA1	73	PXN	74	ZNF263	75	NOS1	76	FLG	77	HDAC2	78	RFX5	79	HUWE1	80	SHC1
81	TBP	82	IRF1	83	AHR	84	DLG1	85	HDAC6	86	HIPK2	87	IRS1	88	PAK2	89	SDC2	90	ID2
91	LAMB1	92	GRB2	93	RUNX2	94	MAP3K3	95	IL16	96	F2	97	COPS5	98	SIRT1	99	TLN1	100	LRP2
101	PRKCE	102	FZD4	103	SMARCA2	104	EPHA2	105	REST	106	GSN	107	GNAL	108	NALCN	109	GNGT1	110	RYR1
111	NEDD4	112	IKBKG	113	RASA1	114	ACAN	115	TTN	116	ITGB1	117	APOB	118	PPP2CA	119	WNT1	120	PIK3R2
121	NFKBIA	122	LRP6	123	PTK2B	124	CSNK2A1	125	PDPK1	126	ANK2	127	RYK	128	CDK1	129	CEBPB	130	ATF2
131	SOD1	132	SIN3A	133	MAPT	134	MAPK3	135	CFTR	136	RXRA	137	RELA	138	ATXN1	139	BTRC	140	HCK
141	KHDRBS1	142	ITGA1	143	GAPDH	144	GABPA	145	LRRK2	146	MAFF	147	OBSCN	148	CALM3	149	INS	150	YY1
151	ACTL6A	152	COL4A1	153	EFTUD2	154	MAFK	155	ABCA1	156	SIX5	157	RYR3	158	TRAF6	159	EXO1	160	PLK1
161	HIST1H3J	162	NAV3	163	IRAK1	164	UCHL5	165	HDAC5	166	ZNF143	167	PRKACB	168	SETDB1	169	CSF2RA	170	HSPA1A
171	CAT	172	SUMO1	173	HDAC3	174	UNC79	175	PPP2R5C	176	DNAH5	177	ANK3	178	NR3C1	179	HIST1H3E	180	PLG
181	RYR2	182	HSPB1	183	UBQLN4	184	KAT5	185	TNFSF10	186	NR2C2	187	JAG1	188	PRKCQ	189	UBC	190	JUND
191	NR4A1	192	TGFBR1	193	COL1A2	194	HSPG2	195	CUL1	196	CTBP2	197	FBN1	198	GSK3B	199	E2F4	200	CDK2
201	PBX3	202	MAPK14	203	YES1	204	IL2RG	205	HSPA4	206	BUB1	207	YWHAZ	208	CEBPD	209	IKBKE	210	VCAN
211	SOCS3	212	HSPA5	213	ITGB3	214	USF1	215	PLEC	216	ATF3	217	LEP	218	RPS6KA2	219	DST	220	SPTBN1
221	CTNNA1	222	HIST1H3I	223	MED1	224	STAT5A	225	SPTAN1	226	CHUK	227	HIST1H3G	228	VCL	229	TAF1	230	TFAP2C
231	IGF2	232	ACTA1	233	ALB	234	NFATC1	235	FOXA2	236	SPI1	237	U2AF2	238	FREM2	239	SREBF2	240	SMARCC1
241	PAX6	242	PIK3R3	243	TFAP2A	244	FOS	245	PTK2	246	UBA52	247	IFNG	248	ISG15	249	NOTCH3	250	BATF
251	NGFR	252	SP1	253	SPTA1	254	PTPN1	255	HNF4G	256	GTF2B	257	TP73	258	TJP1	259	BMP7	260	TYK2
261	BAG3	262	ITPR1	263	ICAM1	264	SMAD7	265	ZAP70	266	HIST1H3C	267	NFYB	268	CSMD1	269	GAB2	270	POLR2A
271	STAT1	272	SMC3	273	USH2A	274	PRKDC	275	ACTA2	276	HDAC4	277	MEF2C	278	IQGAP1	279	BMP2	280	GLI3
281	GRIP1	282	TRAF2	283	ITCH	284	HGS	285	FN1	286	HSPD1	287	ELF1	288	CHD3	289	HTT	290	JUP
291	INSR	292	SYNE1	293	IRS2	294	COL5A1	295	CALM1	296	LAMA1	297	HSPA8	298	SLC2A1	299	MACF1	300	ITGB4
301	GNAI1	302	IL2RB	303	CACNA1A	304	MCL1	305	FLNC	306	PRKCD	307	DLG4	308	NCAM1	309	HNF4A	310	SNCA
311	FYN	312	DMD	313	CDC42	314	SRF	315	MEF2A										

We use SGCD to train and predict on six PPIs. Then, by taking the union of the top 100 predicted cancer driver genes from each PPIs, a list of 315 potential cancer

*Corresponding author: xingyili@nwpu.edu.cn, shang@nwpu.edu.cn

driver genes is obtained, as shown in Table. S1. To further analyze these potential cancer genes, we compare them with two lists of candidate cancer driver genes derived from literature-based sources The first source is the CancerMine [1], a text-mined and regularly updated resource which catalogs drivers, oncogenes and tumor suppressor genes (TSGs) across various cancer types. The second source is a high-confidence gene set collected from the Candidate Cancer Gene Database (CCGD) [2], which includes all published data from transposon-based forward genetic screens for cancer. Overall, approximately 91% (287/315) of the potential driver genes have evidences supporting their association with cancer. Furthermore, among these evidence-supported genes, over 88% (253/287) are supported by CancerMine, over 76% (220/287) are supported by CCGD and over 64% (186/287) are supported by both CancerMine and CCGD. These experimental results further substantiate the strong reliability of the cancer driver genes identified by SGCD.

2. The homophily ratio of PPIs

To assess the level of heterophily in PPIs, we introduce the homophily ratio to determine whether a network exhibits homophilic or heterophilic characteristics [3]. The homophily ratio is calculated as the proportion of neighboring nodes belonging to the same class relative to the total number of neighboring nodes in the graph, and can be defined as follows:

$$h = \frac{1}{|\mathcal{V}|} \sum_{v_i \in \mathcal{V}} \frac{|\{v_j | v_j \in \mathcal{N}_i, Y_j = Y_i\}|}{|\mathcal{N}_i|} \quad (1)$$

where $\mathcal{V}$ is the set of nodes, $\mathcal{N}_i$ is the neighbor set of node v_i, Y_i is label of node v_i. Graphs that exhibit strong homophily are characterized by a high homophily ratio approaching 1, whereas graphs with heterophily (i.e., low or weak homophily) have an edge homophily ratio that tends toward 0 [4].

The homophily ratios of PPIs are shown in Table S2. The results show that PPIs from different databases all exhibit low homophily ratios, indicating that there are a small number of similar nodes among the neighbors of driver genes. This also suggests that there are too many inter-class edges in the PPIs, which leads to the confusion of features between different types of nodes after aggregation, making them indistinguishable and thus affecting the performance of GCNs.

Table S2.: The overview of PPIs

Name	Number of Edges	Number of Nodes	Number of Positive Samples	Number of Negative Samples	Homophily Ratio
CPDB	252,189	13,627	796	2187	0.1568
STRINGdb	336,549	13,179	783	2415	0.1889
MULTINET	109,567	14,398	790	3709	0.1002
PCNet	2,724,724	19,781	859	5483	0.1911
IRefIndex	371,568	17,013	836	4056	0.1678
IRefIndex 2015	91,809	12,129	785	1973	0.1547

3. Acquisition of pan-cancer multiomics data

We collect cancer genomics (mutations and copy number), epigenomics (DNA methylation), and transcriptomics (gene expression) from the cancer genome atlas (TCGA, `https://portal.gdc.cancer.gov/`), encompassing over 29,446 samples across 16 distinct cancer types, including Bladder Urothelial Carcinoma (BLCA), Breast invasive carcinoma (BRCA), Cholangiocarcinoma (CHOL), Colon adenocarcinoma (COAD), Esophageal carcinoma (ESCA), Head and Neck squamous cell carcinoma (HNSC), Kidney renal clear cell carcinoma (KIRC), Kidney renal papillary cell carcinoma (KIRP), Liver hepatocellular carcinoma (LIHC), Lung adenocarcinoma (LUAD), Lung squamous cell carcinoma (LUSC), Pancreatic adenocarcinoma (PAAD), Prostate adenocarcinoma (PRAD), Rectum adenocarcinoma (READ), Thyroid carcinoma (THCA), and Uterine Corpus Endometrial Carcinoma (UCEC). For each gene, we calculate gene mutation rate, copy number aberrations (CNAs), differential DNA methylation rate, and differential gene expression rate across the 16 cancer types:

- Gene mutation rate
  The mutation rate of each gene in a given cancer type is defined as the number of non-silent mutations in that gene, divided by its exonic length. To compute gene mutation rate, we acquire exon lengths genomic annotation data obtained from GENCODE [5]. The gene mutation rate is calculated as:

$$mf_i^c = \frac{1}{|p_c|} \sum_{p \in p_c} F_{p,i} \tag{2}$$

  For cancer type c, P_c is the set of patients and $F_{p,i}$ is the mutation frequency for the sample from the patient p of the gene i.
- CNAs
  Gene-associated CNAs are collected from TCGA data, encompassing both amplifications and deletions, while ultramutated samples from syn1729383 are excluded from our study. The copy number rate for each gene is defined as the total number of times that gene is either amplified or deleted in a specific cohort.
- Differential DNA methylation rate
  DNA methylation data is collected from the Illumina Human Methylation 450K BeadChip for tumor and corresponding tumor-adjacent normal tissue samples. The differential DNA methylation rate is calculated as:

$$dm_i^c = \frac{1}{N_d} \sum_{p \in p_c} \left(\beta_{p,i}^t - \beta_{p,i}^n\right) \tag{3}$$

  where $\beta_{p,i}^t$ and $\beta_{p,i}^n$ are the DNA methylation level for the tumor sample and the normal sample respectively from the patient p of the gene i in cancer type c. N_d is the number of patients who have paired tumor samples and corresponding tumor-adjacent tissue samples (normal samples).
- Differential gene expression rate
  We filter out genes whose number of zero values are more than 10% of the total number of samples to reduce the impact of noise. Subsequently, all the data

are log2-transformed. The differential gene expression rate is calculated as:

$$ge_i^c = \frac{1}{N_e} \sum_{p \in p_c} log_2 \left(\frac{V_{p,i}^t}{V_{p,i}^n} \right) \tag{4}$$

where $V_{p,i}^t$ and $V_{p,i}^n$ are the gene expression level for the tumor sample and the normal sample respectively from the patient p of the gene i in cancer type c. N_e is the number of patients who have both tumor and normal samples in gene expression data.

By concatenating these vectors across all cancer types, we obtain a 64-dimensional feature vector for each gene. Finally, feature-wise min-max normalization is applied to each gene.

4. Drug sensitivity analysis

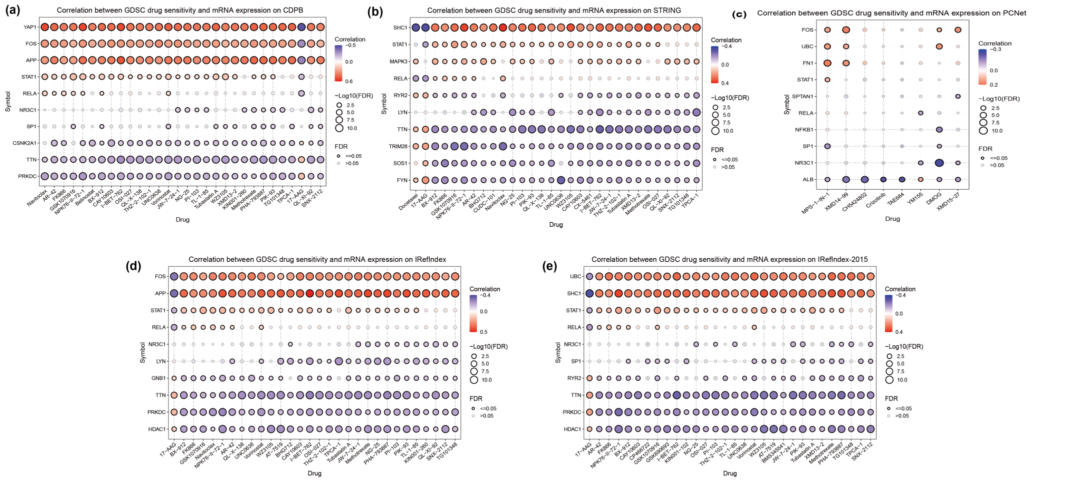

Fig. S1.: Drug sensitivity analysis of SGCD.
(a) Drug sensitivity analysis on CPDB. **(b)** Drug sensitivity analysis on STRINGdb. **(c)** Drug sensitivity analysis on PCNet. **(d)** Drug sensitivity analysis on IRefIndex. **(e)** Drug sensitivity analysis on IRefIndex-2015.

We select the top 10 predicted cancer driver genes in each dataset for Cancer Therapeutics Response Portal (CPTR) drug sensitivity analysis using Gene Set Cancer Analysis (GSCA, `http://bioinfo.life.hust.edu.cn/GSCA`) [6, 7]. Fig. S1 shows the drug sensitivity analysis results for different datasets including CPDB, STRING, PCNet, IRefIndex, and IRefIndex-2015. The results of the drug sensitivity analysis reveal that cancer driver genes identified by SGCD provide crucial insights into potential drug targets, enhancing both the effectiveness and precision of cancer treatments. For example, BCL-2 family inhibitors, such as Navitoclax, have been investigated for their potential as anti-cancer therapies. Navitoclax induces apoptosis in cancer cells by disrupting the interactions of anti-apoptotic proteins[8]. Docetaxel (DTX) is recognized as one of the most potent anticancer agents, with broad applicability across various cancer

treatments[9]. OSI-027, an orally bioavailable compound, has demonstrated anti-cancer activity in multiple cancer cell lines and tumor xenograft models[10]. Vorinostat has also been evaluated in numerous clinical trials for treating a wide range of hematological and solid tumors, including lymphoma, breast cancer, non-small cell lung cancer (NSCLC), glioblastoma multiforme, and head and neck squamous cell carcinomas[11].

5. Gene module dissection in pan-cancer

We employ the model-agnostic approach GNNExplainer[12] to interpret the contribution factors to cancer driver genes within the multi-omics, and further identify the cancer gene modules. After that, we compare the topological structures of cancer gene modules and non-cancer gene modules using graphical metrics, including PageRank, clustering coefficient, degree centrality, and betweenness centrality[13].

- PageRank

The PageRank algorithm can reflect the importance or centrality of nodes in a network, and the PageRank centrality of a node i is calculated as follows:

$$C_{PR}(i) = d\left(\sum_{j\in\Gamma^-(i)} \frac{C_{PR}(j)}{|\Gamma^+(j)|}\right) + \frac{1-d}{n} \tag{5}$$

where $\Gamma^-(j)$ is the set of nodes pointing to node i, $\Gamma^+(j)$ is the set of nodes pointed to by node j, d is a damping factor, and n represents the number of nodes

- Clustering coefficient

Clustering coefficient measures the proportion of closed triangles within a node's local neighborhood. The commonly used local variant of the clustering coefficient is computed as follows:

$$C_u = \frac{|(v_1, v_2) \in \mathcal{E} : v_1, v_2 \in \mathcal{N}(u)|}{\binom{d_u}{2}}. \tag{6}$$

Where the numerator counts the number of edges between the neighbors of node u, $\mathcal{N}(u) = \{v \in \mathcal{V} : (u, v) \in \mathcal{E}\}$ denotes the neighborhood of node u, and the denominator calculates the total number of pairs of nodes in u's neighborhood.

- Degree centrality

Degree centrality is a simple and effective local centrality metric that measures the centrality of a node based on the number of links it has with its neighbors. Degree Centrality can be defined as follows:

$$C_D(i) = \frac{1}{n-1}\sum_{j=1}^{n} a_{ij} \tag{7}$$

where a_{ij} is an element of the adjacency matrix A, indicating the connectivity between

nodes i and j. Specifically, $a_{ij} = 1$ if a link exists between nodes i and j, otherwise $a_{ij} = 0$.

- Betweenness centrality

Betweenness centrality quantifies a node's centrality by calculating the ratio of the number of shortest paths between any pair of nodes that pass through the given node to the total number of shortest paths between that pair. It can be defined as follows:

$$C_B(i) = \sum_{i \neq j \neq k \in V} \frac{|SP_{jik}|}{|SP_{jk}|} \tag{8}$$

where SP_{jk} represents the set of shortest paths between nodes j and k, while SP_{jik} denotes the subset of these shortest paths that pass through node i.

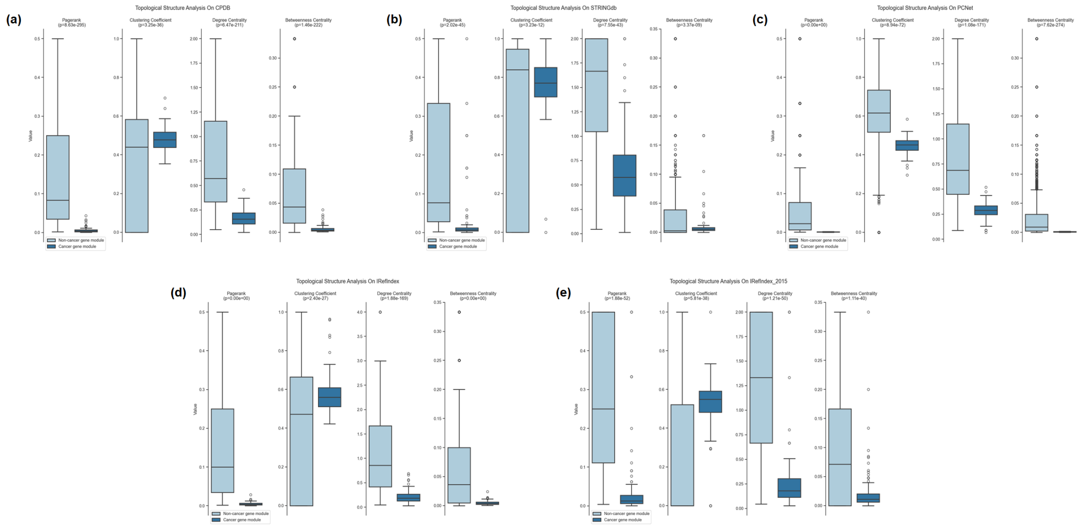

Fig. S2.: Graphical metrics of gene modules
(a) Topological analysis on CPDB. **(b)** Topological analysis on STRINGdb. **(c)** Topological analysis on PCNet. **(d)** Topological analysis on IRefIndex. **(e)** Topological analysis on IRefIndex-2015.

Fig. S2 gives the results of comparison of the topological structures of cancer gene modules and non-cancer gene modules using graphical metrics in different datasets including CPDB, STRING, PCNet, IRefIndex, and IRefIndex-2015, and reveals a striking difference between cancer gene modules and non-cancer gene modules. Specifically, the results indicate significant differences in the topological structures between cancer and non-cancer gene modules. This finding is supported by a highly significant p-value from t-test, which underscores the robustness of the observed difference.

References

[1] J. Lever, E. Y. Zhao, J. Grewal, M. R. Jones, and S. J. Jones, "Cancermine: a literature-mined resource for drivers, oncogenes and tumor suppressors in cancer," Nature methods, vol. 16, no. 6, pp. 505–507, 2019.

[2] K. L. Abbott, E. T. Nyre, J. Abrahante, Y.-Y. Ho, R. Isaksson Vogel, and T. K. Starr, "The candidate cancer gene database: a database of cancer driver genes from forward genetic screens in mice," Nucleic acids research, vol. 43, no. D1, pp. D844–D848, 2015.

[3] Y. Ma, X. Liu, N. Shah, and J. Tang, "Is homophily a necessity for graph neural networks?" arXiv preprint arXiv:2106.06134, 2021.

[4] J. Zhu, Y. Yan, L. Zhao, M. Heimann, L. Akoglu, and D. Koutra, "Beyond homophily in graph neural networks: Current limitations and effective designs," Advances in neural information processing systems, vol. 33, pp. 7793–7804, 2020.

[5] A. Frankish, M. Diekhans, I. Jungreis, J. Lagarde, J. E. Loveland, J. M. Mudge, C. Sisu, J. C. Wright, J. Armstrong, I. Barnes et al., "Gencode 2021," Nucleic acids research, vol. 49, no. D1, pp. D916–D923, 2021.

[6] C.-J. Liu, F.-F. Hu, M.-X. Xia, L. Han, Q. Zhang, and A.-Y. Guo, "Gscalite: a web server for gene set cancer analysis," Bioinformatics, vol. 34, no. 21, pp. 3771–3772, 2018.

[7] C.-J. Liu, F.-F. Hu, G.-Y. Xie, Y.-R. Miao, X.-W. Li, Y. Zeng, and A.-Y. Guo, "Gsca: an integrated platform for gene set cancer analysis at genomic, pharmacogenomic and immunogenomic levels," Briefings in bioinformatics, vol. 24, no. 1, p. bbac558, 2023.

[8] N. N. Mohamad Anuar, N. S. Nor Hisam, S. L. Liew, and A. Ugusman, "Clinical review: Navitoclax as a pro-apoptotic and anti-fibrotic agent," Frontiers in Pharmacology, vol. 11, 2020.

[9] N. Chaurawal and K. Raza, "Nano-interventions for the drug delivery of docetaxel to cancer cells," Health Sciences Review, vol. 7, p. 100101, 2023.

[10] M. Rehan, "An anti-cancer drug candidate osi-027 and its analog as inhibitors of mtor: Computational insights into the inhibitory mechanisms," Journal of Cellular Biochemistry, vol. 118, 2017. [Online]. Available: https://api.semanticscholar.org/CorpusID:1169963

[11] V. K. H. Le, T. P. D. Pham, and D. H. Truong, "Delivery systems for vorinostat in cancer treatment: An updated review," Journal of Drug Delivery Science and Technology, vol. 61, p. 102334, 2021. [Online]. Available: https://www.sciencedirect.com/science/article/pii/S1773224721000150

[12] Z. Ying, D. Bourgeois, J. You, M. Zitnik, and J. Leskovec, "Gnnexplainer: Generating explanations for graph neural networks," Advances in neural information processing systems, vol. 32, 2019.

[13] K. Chi, N. Wang, T. Su, Y. Yang, and H. Qu, "Measuring the centrality of nodes in networks based on the interstellar model," Information Sciences, p. 120908, 2024.
